# Optimizing stimulation parameters: transpalpebral and transbrain electrical stimulation for retinal protection in RCS rats

**DOI:** 10.3389/fcell.2026.1853872

**Published:** 2026-07-17

**Authors:** Nannan Shi, Weidao Zhang, Miaoran Gao, Jiaxian Li, Yamin Li, Kai Xu, Lina Liang

**Affiliations:** 1 Department of Eye Function Laboratory, Eye Hospital China Academy of Chinese Medical Sciences, Beijing, China; 2 Postdoctoral Research Station of China Academy of Chinese Medical Sciences, Beijing, China; 3 Department of Ophthalmology, The First Affiliated Hospital of Yunnan University of Chinese Medicine, Yunnan Provincial Hospital of Traditional Chinese Medicine, Kunming, China

**Keywords:** neurotrophic factors, non-invasive electrical stimulation, photoreceptor apoptosis, retinitis pigmentosa, the Foot Shaoyang Gallbladder meridian

## Abstract

**Introduction:**

Retinitis pigmentosa is a genetically heterogeneous retinopathy causing photoreceptor death and blindness. Electrical stimulation is a promising non-invasive neuroprotective strategy. This study investigated the neuroprotective effects of transpalpebral ES (TpES) and transbrain ES (TbES) delivered at Gallbladder meridian acupoints (GB14, GB16, GB20) in Royal College of Surgeons (RCS) rats, and identified optimal parameters for each modality.

**Methods:**

RCS rats were randomized to control, sham, TpES (200, 400, 600, 800 µA), and TbES (15, 30, 50, 100 Hz) groups. Stimulation was given daily for 14 days from 6 weeks of age. Visual-guided behavior was assessed by open-field test (OFT). Retinal function and morphology were evaluated by electroretinography (ERG), color fundus photography (CFP), spectral-domain optical coherence tomography (SD-OCT), and histopathology. Photoreceptor apoptosis was quantified by terminal deoxynucleotidyl transferase dUTP nick-end labeling (TUNEL). Neurotrophic factors (BDNF, CNTF, bFGF, NGF) and c-Fos were detected by immunofluorescence and digital PCR.

**Results:**

OFT revealed progressive anxiety-like behavior in untreated control rats, though non-visual confounds cannot be excluded. All stimulated groups exhibited stable locomotor activity comparable to sham, with no behavioral abnormalities or systemic adverse effects. All stimulated groups showed stable locomotor activity comparable to sham, with no adverse effects. TpES at 800 µA and TbES at 50 Hz produced the greatest preservation of retinal function and structure, maintaining 3–4 photoreceptor cell layers versus 1–2 layers in controls. TpES at 800 µA reduced the apoptotic index by ∼35%; all TbES frequencies lowered it to a similar range, indicating a frequency-independent anti-apoptotic threshold. Both modalities upregulated BDNF and bFGF; TbES—especially at 50 Hz—additionally upregulated CNTF and NGF, a pattern not seen in TpES groups. Elevated c-Fos confirmed inner retinal neuron activation.

**Conclusion:**

Optimized TpES (800 µA) and TbES (50 Hz) each exert neuroprotective effects, preserving retinal function and structure, reducing apoptosis, and upregulating neurotrophic factors, without behavioral or systemic side effects. These eye-brain ES approaches, grounded in traditional Chinese medicine meridian theory and modern neuromodulation, provide evidence-based parameters for clinical translation and represent a promising synergistic strategy.

## Graphical Abstract

**Figure:**
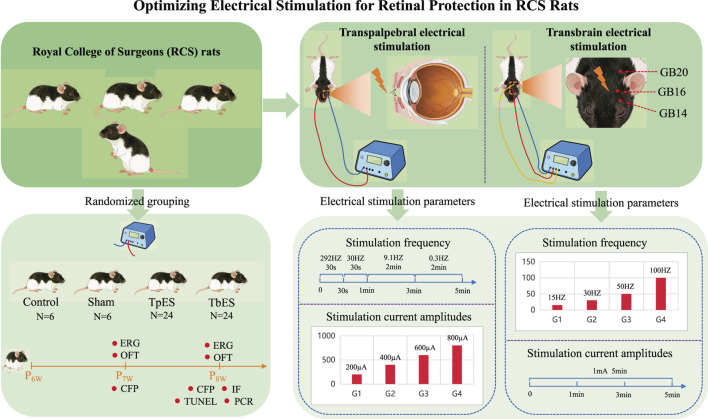

## 1 Introduction

Retinitis pigmentosa (RP) encompasses a group of inherited retinal dystrophies (IRDs) that primarily affect rod and cone photoreceptors, leading to progressive retinal degeneration (Verbakel et al., 2018). With a global prevalence of 1 in 5,000, RP affects over 1.5 million patients worldwide (Liu et al., 2024). The initial symptom of RP is generally nyctalopia, or loss of night vision, followed by a gradual narrowing of visual fields, although the condition can present with a variety of clinical manifestations. Over time, it causes irreversible damage to visual function and significantly reduces patients’ quality of life (O’Neal et al., 2026). While gene and stem cell therapies have made some progress in the field of RP, treatments have been limited to patients with specific genetic backgrounds, and procedures are invasive, with potential tumor growth or retinal damage (Liu et al., 2024; Ma and Shen, 2025). Consequently, there is an urgent need to develop new treatment strategies that are safe, effective and accessible.

RP poses irreversible threats to vision, mainly due to the death of retinal neurons, a permanent change characterized by the loss of neuronal cell bodies, axons, and dendrites (Oshitari, 2024). Neuroprotective strategies aim to increase retinal neuron survival and preserve visual function (Pardue and Allen, 2018). Electrical stimulation (ES) therapies are becoming popular for rehabilitation, ranging from neuromuscular stimulation to strengthen muscles and synaptic connections to neurostimulation devices that modulate neuronal activity and provide therapeutic effects. For vision, two types of neurostimulation are being used: prosthetics to activate visual circuits by directly stimulating retinal neurons or the visual cortex, and low-level ES that provides neuroprotection. Presently, there are eight routes by which ES has been delivered to patients to improve vision: transpalpebral, transorbital, transcorneal, subretinal, epiretinal, and transchoroidal approaches, as well as direct stimulation of the optic nerve or brain (Sehic et al., 2016). Transpalpebral ES (TpES) applies a microcurrent through the eyelid skin, which is effectively transmitted to the posterior segment of the eye, avoiding complications associated with corneal irritation. Therefore, noninvasive TpES is emerging as a promising and versatile tool for modulating neuronal activities and functional rehabilitation.

Visual perception is not confined to the retina; following phototransduction, neural signals are relayed via the optic nerve and lateral geniculate nucleus to the primary visual cortex, where conscious vision is ultimately formed (Wandell et al., 2007; Cuenca et al., 2014). Transbrain ES (TbES), which delivers low-intensity current through the scalp to modulate cortical and subcortical neuronal activity, has emerged as a non-invasive approach to enhance visual cortical plasticity and promote neuroprotection along the entire retinocortical axis (Park and Thompson, 2024; Duan et al., 2025). Meanwhile, research on acupuncture has also demonstrated that stimulating distal acupoints related to vision (such as UB60 and GB37) can selectively activate visual processing regions of the brain, including the occipital cortex. This suggests that acupoint stimulation may influence the activity of the visual center via specific neural pathways (Li et al., 2003; Kong et al., 2009). Notably, in traditional Chinese medicine (TCM), the Foot Shaoyang Gallbladder meridian (足少阳胆经) encompasses a series of acupoints anatomically aligned with the visual pathway, from the periorbital region to the occipital cortex. Among these, Yangbai (GB14), located directly above the orbital margin in the frontal region, Muchuang (GB16), situated on the lateral scalp overlying visual association cortex, and Fengchi (GB20), found in the suboccipital depression adjacent to the occipital visual cortex, corresponding to key brain regions involved in modern visual attention, information integration and primary processing (China Association of Acupuncture and Moxibustion, 2025). The integration of TbES with TCM meridian theory thus provides a physiologically coherent, multi-target therapeutic framework that addresses neuroprotection across the entire visual system, from photoreceptors to visual cortex.

Rats from the Royal College of Surgeons (RCS) are commonly used as an animal model in RP research. These rats exhibit impaired phagocytic function in the retinal pigment epithelium due to an autosomal recessive mutation in the MERTK gene (Gal et al., 2000). In this study, we thoroughly investigated the neuroprotective effects of different current amplitudes (200–800 µA) for TpES and stimulation frequencies (15–100 Hz) for TbES applied at Gallbladder meridian acupoints (GB14, GB16, and GB20) in the RCS rat model of inherited photoreceptor degeneration. Retinal function, structural integrity, photoreceptor apoptosis, and the expression of key neuroprotective factors were systematically assessed. The results aim to establish evidence-based criteria for parameter optimization and to provide a quantitative basis for developing future combined eye-brain ES protocols for the clinical management of RP.

## 2 Materials and methods

### 2.1 Animals

Male and female RCS rats, with a body weight range of 150–200 g, were obtained from a certified Institute of Medical Laboratory Animals, Chinese Academy of Medical Sciences, Beijing, China. All animals were housed under a 12-h light/dark cycle at a controlled ambient temperature (22 °C ± 2 °C) with standard chow diet in a specific pathogen–free facility at the Eye Hospital, China Academy of Chinese Medical Sciences. ES interventions were initiated at 6 weeks of age, a stage characterized by reduced outer nuclear layer (ONL) thickness. The ONL thinned uniformly across all four quadrants, with significant loss observed in numerous regions of the posterior pole retina—closer to real-world disease management scenarios (Ryals et al., 2017). All procedures were performed in accordance with the Association for Research in Vision and Ophthalmology (ARVO) Statement for the Use of Animals in Ophthalmic and Vision Research and the Animal Research: Reporting of *In Vivo* Experiments (ARRIVE) guidelines. The sample size for each group was determined based on the exploratory parameter-optimization design of the study, the number of stimulation conditions to be evaluated, and the ethical imperative to minimize animal use in accordance with the 3R principle. The experimental protocol was reviewed and approved by the Medical Ethics Committee of Eye Hospital, China Academy of Chinese Medical Sciences (Approval No. YKEC-KT-2024-066-P003).

### 2.2 Experimental design

RCS rats were randomly assigned to the following groups: untreated rats represents the natural course of retinal degenerative diseases in RCS (Control group); a sham stimulation group (Sham group), in which animals underwent identical handling and electrode placement procedures at all stimulation sites without current delivery, thereby serving as an anaesthesia and handling-matched control that isolates the effect of current delivery from that of repeated isoflurane exposure and electrode placement. A microcurrent stimulus generator (Eyescure (Suzhou) BE Co., Ltd.) was employed to deliver TpES. Han’s acupoint nerve stimulator (Nanjing Jisheng Medical Technology Co., Ltd.) was utilized to apply TbES. Four TpES groups receiving current amplitudes of 200, 400, 600, and 800 μA, which comprised of a biphasic pulse with a frequency of 292 Hz for 30 s, 30 Hz for 30 s, 9.1 Hz for 2 min, and 0.3 Hz for 2 min; and four TbES groups receiving stimulation at constant amplitude continuous wave frequencies of 15, 30, 50, and 100 Hz at a fixed amplitude of 1 mA. All stimulated groups and the sham group received daily anaesthesia of identical duration, the untreated control group was anaesthetised only during terminal functional and imaging assessments. Detailed stimulation safety parameters (electrode area, charge per phase, charge density) are provided in Supplementary Table S1. For TpES, surface electrodes were placed at the midpoints of the upper and lower eyelids bilaterally, and stimulation was delivered at the assigned current amplitude for 5 min. For TbES, stainless steel needle electrodes were inserted subcutaneously at three standardized Gallbladder meridian acupoints bilaterally(China Association of Acupuncture and Moxibustion, 2025): Yangbai (GB14, 3 mm directly superior to the eyeball center), Muchuang (GB16, at the lateral one-third point of the line connecting Yangbai and Fengchi), and Fengchi (GB20, in the depression inferior to the occipital ridge), delivering stimulation at the assigned frequency for 5 min a day. Stimulation sessions were administered once daily for 14 consecutive days beginning at postnatal week 6.

### 2.3 Open-field test

Locomotor activity and anxiety-related behavior were assessed using an open-field test (OFT) to monitor potential systemic effects of repeated electrical stimulation and to provide a general index of visual-guided behavior. Animals were evaluated at 7 days and 14 days after the initiation of the stimulation protocol. Send the rats to the test room 1 h in advance to fit the experimental environment. The area was thoroughly cleaned with 1% ethanol between individual trials to maintain a consistent smell environment. Each rat was individually placed in the center of a square open-field area and allowed to explore freely for 5 min. The open field test area had dimensions of 100 cm (L) × 100 cm (W) × 40 cm (H). Behavioral parameters, including total distance traveled, velocity, Center Cumulative Duration, and Zone transition Center-point, were automatically tracked and quantified using EthoVision XT 16 software.

### 2.4 Electroretinography

The experimental animals completed 12 h of standardized dark adaptation. General anesthesia was induced and maintained with isoflurane (Shenzhen Ruiwode Life Science and Technology Co., LTD), using approximately 2% isoflurane in 0.2–0.5 L/min O_2_. After adequate pupil dilation with compound tropicamide eye drops, the eye surface was anesthetized with hydrochloric obucaine eye drops. The entire procedure was carried out under weak red light (wavelength >620 nm). ERGs were recorded using a Celeris Rodent ERG system (Diagnosys LLC). The rats were secured on the operating table to maintain head stability. Two circular corneal contact electrodes were placed in the center of each eye. After confirming the correct electrode connection, we lubricated the electrodes with a sodium carboxymethyl cellulose solution to achieve optimal contact. Data acquisition was performed using Espion software (Diagnosys). The stimulus protocols used were designed to reflect the International Society for Clinical Electrophysiology of Vision (ISCEV) standards for ERG (Robson et al., 2022). For each animal, ERG was recorded from the right eye only. Three animals were used per group, yielding n = 3 eyes per group for statistical analysis.

### 2.5 *In Vivo* imaging

Structural retinal integrity was assessed non-invasively by color fundus photography (CFP) and spectral-domain optical coherence tomography (SD-OCT) at 7 and 14 days after stimulation onset. Animals were anesthetized as described above, and pupils were fully dilated with topical 1% tropicamide prior to imaging. CFP was performed using an OPTO-RIS retinal imaging system (Beijing Xinlian Optoelectronic Technology Co., Ltd., Beijing, China) to document gross retinal appearance. SD-OCT cross-sectional imaging was performed using an Envisu R2210 OCT system (Bioptigen, Inc., Morrisville, NC, United States) with a rodent contact lens adaptor. Total retinal thickness was measured along the ventral–dorsal meridian, starting 200 μm from the optic nerve head and extending 1,000 μm toward both the ventral and dorsal sides. Measurements from both sides were averaged prior to statistical analysis. The cornea was kept moist by frequent application of sodium hyaluronate eye drops throughout the entire imaging session. After the experiment, erythromycin eye ointment was applied to protect the cornea. All imaging was performed on the right eye of each animal. Three animals per group were used, resulting in n = 3 eyes per group for retinal thickness quantification.

### 2.6 Histological analysis

Animals were euthanized 14 days post-electrical stimulation by overdose of anesthesia, and the eyes were immediately enucleated and dissected to remove excess conjunctiva and extraocular muscles. Whole eyes were fixed in Davidson’s solution for 3 h at room temperature. After fixation, the anterior segment and vitreous body were carefully removed. The remaining posterior eyecups were rinsed briefly in cold phosphate buffered saline (PBS), blotted dry, and processed for dehydration. Tissues were dehydrated through an automated tissue processor. Following dehydration, eyes were embedded in paraffin with the optic nerve oriented parallel to the long edge of the embedding cassette. Serial sections of 4 μm thickness were cut along the optic nerve axis. After drying, sections were stored at room temperature until staining. Briefly, after deparaffinization in xylene (two changes, 10 min each) and rehydration through a graded ethanol series (100%i, 100%ii, 95%i, 95%ii, 85%; 5 min each), sections were washed in distilled water for 5 min. Nuclei were stained with Harris hematoxylin (Sigma-Aldrich, St. Louis, MO, United States) for 5 min at room temperature, followed by a brief rinse in tap water for 5 min. Differentiation was performed in 1% acid alcohol (70% ethanol containing 1% concentrated HCl) for 3–6 s, followed by a 5 min tap water rinse. Sections were then treated with 0.2% ammonia water for 30 s to blue, followed by a 5 min tap water rinse. Sections were then counterstained with 1% eosin Y solution (Sigma-Aldrich) for 2 min. After dehydration through a graded ethanol series (75%, 95%i, 95%ii, 100%i, 100%ii, 100%iii; 1 min each) and clearing in xylene (three changes, 1 min each), sections were mounted with neutral mounting medium (Boster Biological Technology, Wuhan, China). Whole-slide imaging was performed using a Pannoramic MIDI II digital slide scanner (3DHISTECH Ltd., Budapest, Hungary), and digital images were captured and stored for analysis. The thickness of the ONL was quantified and averaged from five non-consecutive sections per eye at standardized eccentricities of 500 μm from the optic disc using ImageJ (NIH). One eye per animal was processed for histology. Three animals per group were used, resulting in n = 3 eyes per group for ONL thickness quantification.

### 2.7 TUNEL assay

Rats were euthanised 14 days post-electrical stimulation. Photoreceptor apoptosis was evaluated at 14 days using the terminal deoxynucleotidyl transferase dUTP nick-end labeling (TUNEL). Eyes were dissected and fixed in 4% paraformaldehyde (PFA) at room temperature for 30 min. The anterior segment and vitreous were then removed, and the remaining eyecup section was placed in 4% PFA at 4 °C for overnight fixation. Specimens were then transferred to 30% sucrose solution for dehydration, followed by final transfer to optimal cutting temperature (OCT) for cryopreservation using liquid nitrogen. Cryosections of 8 μm thickness were prepared along the longitudinal axis of the optic nerve using a cryostat microtome. For TUNEL staining (One Step TUNEL Apoptosis Assay Kit, Beyotime, Shanghai, China), procedures were conducted according to the manufacturer’s instructions. The TUNEL reaction mixture was prepared by mixing TdT and dUTP in a 1:9 ratio, then incubated with tissue for 2 h at 37 °C in a dark, humidified chamber. Following incubation, the samples were incubated at room temperature in DAPI (4′,6-diamidino2-phenylindole, Sigma) for 5 min. Fluorescence images were acquired using a laser-scanning confocal microscope (Nikon). One eye per animal was used for TUNEL staining. Five animals per group were used for this assay, yielding n = 5 eyes per group. TUNEL-positive cells were quantified in three non-consecutive sections per eye and averaged. TUNEL-positive cells were directly calculated with ImageJ software on the basis of the method described previously (De et al., 2015).

### 2.8 Immunofluorescence staining

IF staining was performed on retinal cryosections (8 µm) to evaluate the expression and localization of BDNF and c-Fos. Sections were retrieved from −20 °C, warmed at 57 °C for 10 min, and rehydrated in PBS (2 × 15 min) on a rocking shaker. After delineation with a hydrophobic barrier pen, sections were permeabilized with 1% Triton X-100 in PBS for 30 min at room temperature, followed by blocking with 5% BSA at 37 °C for 30 min. Sections were then incubated overnight at 4 °C with rabbit monoclonal anti-BDNF (1:500; ab108319; Abcam) and mouse monoclonal anti-c-Fos (1:500; ab208942; Abcam) diluted in 0.25% Triton X-100. After three PBS with tween washes (15 min each), Rhodamine labeled secondary antibodies (goat anti-rabbit ZF-0316 and goat anti-mouse ZF-0313, 1:100 in PBS) were applied for 2 h at room temperature in the dark. Nuclei were counterstained with DAPI (Boster Biological Technology, Wuhan, China. AR1177, 1:100, 5 min), and sections were mounted with anti-fluorescence attenuation mounting solution and stored at −20 °C protected from light. IF was performed on the same eyes used for TUNEL staining (single eye from each animal, n = 3 eyes per group). Fluorescence images were acquired by confocal microscopy (Nikon) under uniform settings.

### 2.9 Digital PCR (dPCR)

Retinas were dissected from the single eye of each animal. Three biological replicates (each from a different animal) were used per group. Total RNA was extracted from freshly dissected retina at 14 days using TRIzol reagent (RNeasy Plus Universal Tissue Mini Kit 50, QIAGEN) according to the manufacturer’s protocol. RNA concentration was determined using an ultramicro spectrophotometer (Thermo Fisher Scientific). One-step reverse transcription dPCR was performed on a QIAcuity One digital PCR platform (QIAGEN) using the QIAcuity OneStep Advanced EvaGreen Kit (5 mL; QIAGEN) and QIAcuity Nanoplate 8.5k 96-well (QIAGEN). Reactions were assembled at room temperature according to the manufacturer’s instructions. The primers were synthesized by Beijing Ethelick Technology Co., Ltd., and the sequences of the primers are shown in the Table 1. The reaction mix was thoroughly mixed, transferred to the nanoplate, sealed, and placed into the QIAcuity instrument. The dPCR cycling program consisted of reverse transcription at 50 °C for 40 min, RT enzyme inactivation at 95 °C for 2 min, followed by 40 cycles of denaturation at 95 °C for 15 s, annealing at 58 °C for 15 s and extension at 72 °C for 15 s, with a final cooling step at 40 °C for 5 min. Fluorescence signals were detected using the Green channel with an exposure duration of 200 ms and a gain of 5. Absolute copy numbers per microliter of reaction were calculated using QIAcuity Suite software 2.5.0. Then, the target gene is normalized and the relative quantification is calculated. Multiplicative change = (mRNA of target gene/mRNA of β-actin)/(mRNA of control group/mRNA of β-actin). Each sample was measured in technical duplicate, and three biological replicates per group were included.

**TABLE 1:** Primer sequences of dPCR.

Primer name	Sequence
β -actin- forward	TGC​TAT​GTT​GCC​CTA​GAC​TTC​G
β -actin- reverse	GTT​GGC​ATA​GAG​GTC​TTT​ACG​G
CNTF- forward	GCG​ACT​CCA​AGA​GAA​CCT​CC
CNTF- reverse	GGC​ATC​CCA​TCA​GCC​TCA​TT
BDNF- forward	GCG​GCG​TGC​AAA​TTG​GAT​TA
BDNF- reverse	CTG​CGC​CCT​AGC​ACA​AAA​AG
b-FGF- forward	TGC​TCT​AGG​GGA​CTG​GAG​ATT
b-FGF- reverse	GAA​CTG​AAC​TGG​GGA​GGG​AT
NGF- forward	AGAGAGCGCCTGGAGCC
NGF- reverse	ACC​ACA​GGC​CAA​AAC​TCT​GT
c-Fos- forward	TAC​TAC​CAT​TCC​CCA​GCC​GA
c-Fos- reverse	GCT​GTC​ACC​GTG​GGG​ATA​AA

### 2.10 Statistical analysis

All data are expressed as mean ± standard deviation (SD) unless otherwise specified. Statistical analyses were performed using GraphPad Prism software (version 10.0; GraphPad Software, San Diego, CA, United States). For multi-group comparisons, one-way analysis of variance (ANOVA) followed by Tukey’s *post hoc* multiple comparisons test was applied when data met assumptions of normality (Shapiro–Wilk test) and homogeneity of variance (Levene’s test). Non-parametric data were analyzed by the Kruskal–Wallis test with Dunn’s multiple comparisons. A p-value of <0.05 was considered statistically significant. Because TpES and TbES differ in their physical stimulation parameter, electrode type, and site of delivery, they were not treated as arms of a single dose–response continuum. Accordingly, statistical contrasts for parameter optimization were performed within each modality against the shared untreated Control and Sham groups, and no cross-modality equivalence between the two optimal conditions was tested.

## 3 Results

### 3.1 Transpalpebral and transcranial ES do not impair locomotor behavior or induce systemic side effects in RCS rats

**FIGURE 1:**
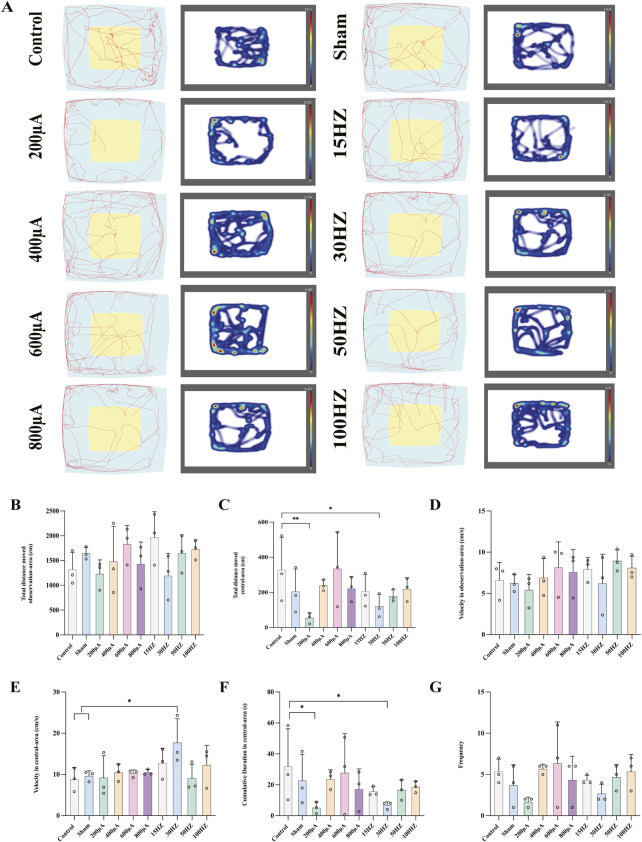
Effects of TpES and TbES on open field test behavior in RCS rats at 7 and 14 days post-stimulation. **(A)** Representative movement trajectory plots (left) and heatmaps (right) of Control, Sham, and electrically stimulated groups (TpES: 200, 400, 600, 800 μA; TbES: 15, 30, 50, 100 Hz), illustrating spatial exploration patterns within the open field area. The yellow zone denotes the central area and the surrounding region represents the observation area. Warmer colors in heatmaps indicate greater time spent in that location. **(B)** Total distance moved in the observation area at 7 days. **(C)** Total distance moved in the central area at 7 days. **(D)** Mean velocity in the observation area at 7 days. **(E)** Mean velocity in the central area at 7 days. **(F)** Center-point cumulative duration at 7 days. **(G)** Zone transition frequency to the central area at 7 days. The horizontal brackets indicate that the grouped items within are statistically different from the other group. Data are presented as mean ± SD (n = 3). *P < 0.05, **P < 0.01.

To monitor potential systemic effects of repeated stimulation and to provide a general index of visual-guided behavior, locomotor activity and exploration were evaluated by OFT at 7 and 14 days after the onset of ES. Representative trajectories and heatmaps showed that animals in every group adopted the periphery-dominant exploration pattern typical of rodents in an open field, circulating mainly within the observation area while making intermittent excursions into the central zone (Figure 1A). Importantly, no abnormal locomotor signatures—circling, freezing, tremor, or seizure-like hyperactivity—were observed in any TpES (200–800 µA) or TbES (15–100 Hz) group, indicating that neither modality produced overt neurological or systemic toxicity.

Gross locomotor capacity was preserved across the stimulated groups. At 7 days, neither the total distance moved nor the mean velocity in the observation area differed significantly between any ES group and the sham group (Figures 1B,D), and the same held for mean velocity in the central area, where only isolated pairwise differences emerged—most notably a transiently higher central-area velocity in the 30 Hz group than in the 50 Hz group (Figure 1E). These patterns persisted at 14 days: aside from the reduced overall activity of the 200 μA and 30 Hz conditions described below, the observation-area distance and velocity of the stimulated groups remained comparable to sham (Supplementary Figure S1). Thus, the stimulation protocols did not compromise basic motor function or general activity levels.

The significant group differences that did arise were confined to metrics of central-zone exploration, occurring selectively in two stimulation conditions. Compared with the control and/or sham groups, the 200 µA TpES group and the 30 Hz TbES group exhibited significantly reduced central-area engagement at 7 days, including lower total distance travelled in the central area (Figure 1C, *P* < 0.05), shorter cumulative duration at the center point (Figure 1F; *P* < 0.05), comparable reductions were confirmed at 14 days (Supplementary Figure S1). Because the total distance moved and mean velocity in the observation area of these same animals were not significantly different from sham at 7 days (Figures 1B,D), the diminished central exploration reflects a more periphery-restricted spatial strategy rather than a generalised locomotor impairment.

In marked contrast to the treated animals, untreated control rats exhibited a progressive change in open-field behavior over the observation period (Figures 1B,F,G and Supplementary Figure S1): mean total distance in the observation area increased by 38.9% (from 1,319.2 ± 340.9 cm to 1832.0 ± 440.5 cm), center cumulative duration increased by 80.0% (from 31.75 ± 24.49 s to 57.13 ± 17.76 s), and zone transition count doubled (from 5.33 ± 1.53 to 10.67 ± 1.15) between 7 and 14 days—an evolving exploration profile consistent with the vision-loss phenotype that develops in this model. Collectively, these results demonstrate that transpalpebral and transcranial ES delivered at the Gallbladder-meridian acupoints are well tolerated, preserving normal locomotor function and general activity without inducing behavioral abnormalities or systemic adverse effects, thereby establishing the behavioral safety of both modalities prior to assessment of their retinal effects.

### 3.2 Optimal parameters of TpES and TbES improve retinal function in RCS rats

**FIGURE 2:**
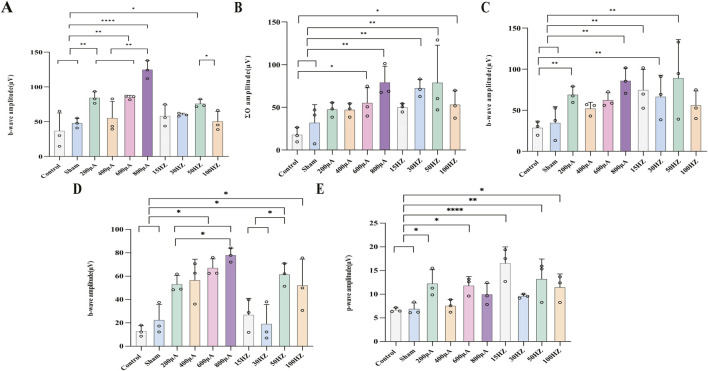
ERG response amplitudes at 7 days post-treatment across all experimental groups. **(A)** Scotopic rod b-wave amplitude. **(B)** Oscillatory potentials (OPs, expressed as ΣO amplitude). **(C)** Maximal mixed response b-wave amplitude. **(D)** Photopic cone b-wave amplitude. **(E)** 10 Hz flicker p-wave amplitude. The horizontal brackets indicate that the grouped items within are statistically different from the other group. Data are presented as mean ± SD (n = 3). *P < 0.05, **P < 0.01.

Full-field ERG was performed at 7 days, 14 days after the completion of ES therapy to evaluate retinal functional changes. At 7 days, the 800 µA group in TpES exhibited the highest amplitudes across all parameters, and was significantly better than control and sham groups for scotopic rod b-wave (124.70 ± 13.01 µV), maximal mixed response (85.83 ± 15.53 µV), oscillatory potentials (OPs) (79.12 ± 19.15 µV), and photopic cone b-wave (78.03 ± 6.04 µV) (Figures 2A–D). In the TbES paradigm, the 50 Hz group provided the best functional outcomes. Compared to control and sham groups, it significantly increased scotopic rod b-wave amplitude (75.68 ± 6.04 µV), maximal mixed response (88.89 ± 47.24 µV), and OPs (78.73 ± 43.99 µV), as well as a comparably elevated photopic cone b-wave amplitude (61.44 ± 9.75 µV) (Figures 2A–D). With respect to the 10 Hz flicker P-wave, the 200 µA group in TpES (12.23 ± 2.94 µV) and the 15 Hz group in TbES (16.54 ± 3.46 µV) yielded the highest amplitudes, substantially exceeding those of both the model (6.66 ± 0.47 µV) and sham (6.89 ± 1.27 µV) groups (Figure 2E).

At 14 days post-treatment, ERG amplitudes in all ES groups remained superior to those of the model and sham groups across most measured parameters. The 800 µA group in TpES showed a numerical increase but not significantly different from scotopic rod b-wave (45.45 ± 7.65 µV), maximal mixed response (43.02 ± 11.03 µV),and 10 Hz flicker (9.75 ± 5.199 µV) (Supplementary Figure S2). However, it maintained significantly higher amplitudes photopic cone b-wave (36.93 ± 11.51 µV) than control (14.43 ± 10.54) and sham groups (24.45 ± 7.97). The 50 Hz group maintained numerically higher mean values but statistical significance against control was lost for maximal mixed response (44.99 ± 40.47 µV). Notably, 50 Hz group exhibited the highest amplitudes and still significantly preserved scotopic rod b-wave (53.75 ± 27.78 µV), OPs (75.52 ± 49.75 µV), and photopic cone b-wave (60.71 ± 9.99 µV) relative to the control group (Supplementary Figure S2). Besides, the 600 µA group in TpES yielded the highest OPs amplitude (34.54 ± 3.48 µV) relative to the control group (14.06 ± 1.84 µV, *P* > 0.05), while the 100 Hz group in TbES produced the highest 10 Hz flicker P-wave amplitude (10.63 ± 10.10 µV) compared to the control group (6.37 ± 1.46 µV, *P* > 0.05). Throughout both time points, the control and sham groups maintained consistently low ERG amplitudes, further supporting the efficacy of ES in preserving retinal function.

Representative ERG waveforms for scotopic responses, OPs, and photopic responses at 7 and 14 days are shown in Supplementary Figures S2F,G. At 7 days, the 800 µA TpES and 50 Hz TbES groups exhibited clearly discernible scotopic b-waves, OPs, and photopic b-waves, with amplitudes substantially higher than those of the control and sham groups, which showed nearly flat or severely attenuated traces. At 14 days, all groups displayed marked reductions in all waveform components. Nevertheless, the 800 μA and 50 Hz groups still retained measurable responses across all three recording conditions, whereas the control and sham traces were almost extinguished. These qualitative observations are consistent with the quantitative ERG data reported above. Collectively, these findings demonstrate that ES, particularly at 800 µA (TpES) and 50 Hz (TbES), confers progressive and sustained improvements in both rod- and cone-mediated retinal responses, with functional benefits continuing to accrue between 7 and 14 days post-treatment.

### 3.3 ES at optimal parameters attenuates retinal structural degeneration in RCS rats

**FIGURE 3:**
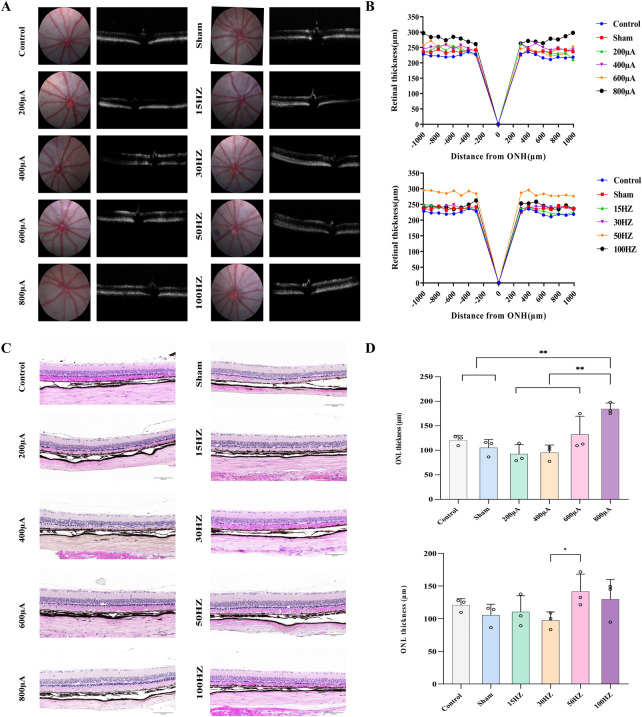
Retinal morphology in RCS rats following ES at varying parameters. **(A)** Representative CFP (left) and SD-OCT cross-sectional images (right) of TpES groups (Control, 200, 400, 600, 800 µA) and TbES groups (Sham, 15, 30, 50, 100 Hz) at 7 days post-stimulation. **(B)** Quantitative retinal thickness along the ventral–dorsal meridian for TpES (upper) and TbES (lower) groups at 7 days post-stimulation. **(C)** Representative HE-stained retinal cross-sections from TpES (left) and TbES (right) groups at 14 days post-stimulation. **(D)** Quantitative ONL thickness across TpES (upper) and TbES (lower) groups at 14 days post-stimulation. The horizontal brackets indicate that the grouped items within are statistically different from the other group. Data are presented as mean ± SD (n = 3). *P < 0.05, **P < 0.01.

The CFP revealed retinal vascular tortuosity and dilation in the fundus of rats from all groups 7 days after ES, accompanied by scattered, patchy, minute pigment spots in the peripheral region (Figure 3A). At 14 days post-stimulation, retinal vascular tortuosity and dilation were markedly more pronounced in all groups compared to the 7 days assessment. This was accompanied by an increase in the number and fusion of pigmented spots in the peripheral regions, predominantly distributed in the mid-periphery and presenting as irregular patches (Supplementary Figure S3). These CFP findings were evaluated qualitatively; no obvious differences between groups were observed that would permit reliable scoring or formal statistical comparison. Therefore, statistical testing was not performed. SD-OCT cross-sectional imaging corroborated these findings: control and sham retinas displayed marked thinning and loss of laminar architecture. while the 800 µA TpES and 50 Hz TbES groups maintained more discernible retinal layering (Figure 3A). Quantitative analysis of total retinal thickness along the ventral–dorsal meridian (200–1,000 µm from the optic nerve head, ONH) demonstrated a current-amplitude-dependent effect of TpES. The 800 µA group exhibited the greatest preservation of retinal thickness at nearly all measured eccentricities compared with the control and sham groups (Figure 3B). Among TbES groups, the 50 Hz condition produced the highest retinal thickness within the TbES paradigm (Figure 3B).

### 3.4 ES reduces photoreceptor apoptosis and preserves outer nuclear layer integrity

#### 3.4.1 TpES and TbES significantly increase outer nuclear layer thickness in RCS rats

To evaluate the structural changes in the retina after ES, HE staining was performed on retina from RCS rats at 14 days post-stimulation. In line with the progressive nature of retinal degeneration in this model, untreated control rats exhibited marked thinning of the ONL and severe disruption of photoreceptor inner and outer segments (IS/OS), indicating advanced photoreceptor loss (Ryals et al., 2017). When rats became 8 weeks, photoreceptors of most groups were nearly completely absent, leaving only 1–2 cell layers (Figure 3C). In contrast, rats treated with optimized ES parameters—particularly the TpES 800 µA and TbES 50 Hz groups—demonstrated notable preservation of ONL integrity and better-defined IS/OS structures compared to sham and control groups. The ONL in these groups was significantly thicker, about 3–4 cell layer and the retinal laminar architecture was better maintained, suggesting a neuroprotective effect of ES on photoreceptor cells. As shown in Figure 3D, statistical comparisons confirmed that the ONL thickness in the 800 µA (184.67 ± 11.88 µm) was significantly greater than that in the control (120.80 ± 9.77 µm), sham (105.47 ± 16.54 µm) and all other treated groups in TpES (*P* < 0.01). For TbES, the 50 Hz (141.73 ± 26.46 µm) group showed a numerical increase in ONL thickness compared to the control and sham groups; however, these differences did not reach statistical significance (*P* > 0.05). Notably, the 50HZ group exhibited significantly greater than that in the 30 HZ group (97.33 ± 13.01 µm) (*P* < 0.05). Low current intensity and suboptimal amplitude ES did not produce a similar protective effect. These results underscore the importance of parameter optimization in achieving meaningful histological preservation in the degenerating retina.

#### 3.4.2 ES markedly reduces the number of apoptotic photoreceptors

**FIGURE 4:**
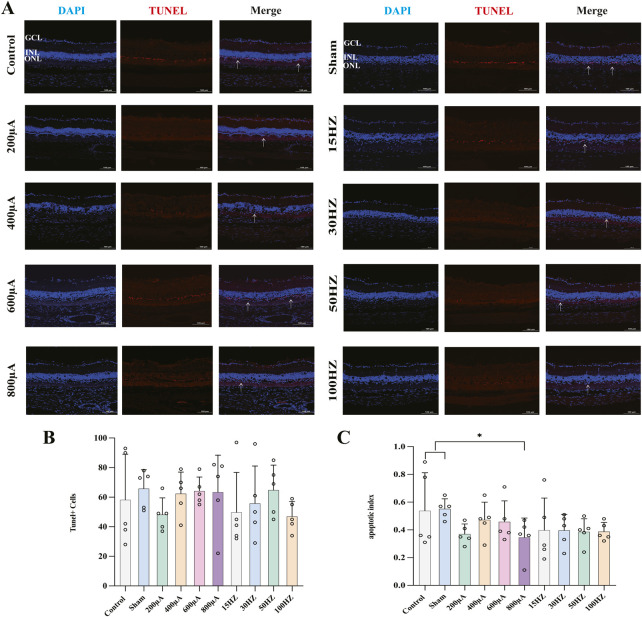
TUNEL staining of photoreceptor apoptosis in RCS rat retinas at 14 days post-stimulation. **(A)** Representative DAPI (left), TUNEL (medium) and merge (right) of all experimental groups (Control, Sham, TpES: 200, 400, 600, 800 μA; TbES: 15, 30, 50, 100 Hz) at 14 days post-stimulation. **(B)** Quantification of TUNEL positive cells per retinal section across all experimental groups. **(C)** Apoptotic index (ratio of TUNEL positive to total DAPI stained nuclei in the ONL) across all groups. The white arrow indicates a positive expression signal. The horizontal brackets indicate that the grouped items within are statistically different from the other group. Data are presented as mean ± SD (n = 5). GCL: ganglion cell layer; INL: inner nuclear layer; ONL: outer nuclear layer. *P < 0.05.

To further investigate the cellular mechanisms underlying ES mediated neuroprotection, TUNEL staining was performed to assess photoreceptor apoptosis in retinal sections at 14 days post-stimulation (Figure 4A).TUNEL positive cells were predominantly localized within the ONL, confirming ongoing photoreceptor degeneration in this model. Quantitative analysis of TUNEL positive cells per retinal section revealed differential anti-apoptotic effects across ES parameters. Among TpES groups, the 200 μA group exhibited a notably reduced number of TUNEL positive cells compared with the control and sham groups, whereas the 400–800 μA groups displayed TUNEL positive cell counts broadly comparable to those of the untreated controls. However, none of them were significantly different from control or sham groups (*P* > 0.05). Within the TbES paradigm, both the 15 Hz and 100 Hz groups demonstrated reduced TUNEL positive cell counts relative to controls, while the 30 Hz and 50 Hz groups remained at levels approximating those of the control and sham groups (Figure 4B). Similarly, none of them were significantly different from untreated groups (*P* > 0.05).

To further quantify the extent of apoptosis, the apoptotic index—defined as the ratio of TUNEL positive cells to the total number of DAPI stained nuclei within the ONL—was calculated for each group (Figure 4C). The control (0.54) and sham (0.55) groups displayed the highest apoptotic indices, reflecting the ongoing photoreceptor degeneration characteristic of the RCS model. Among TpES conditions, the 800 μA group demonstrated the lowest apoptotic index (0.35), representing an approximately 35% reduction relative to control and sham groups (*P* < 0.05), followed by the 200 μA group (0.37, *P* > 0.05). The 400 μA and 600 μA groups yielded intermediate reductions in apoptotic index (0.47 and 0.46 respectively, *P* > 0.05). Within the TbES groups, all tested frequencies (15, 30, 50, and 100 Hz) consistently reduced apoptotic indices to 0.39–0.40, but none were significantly different from control or sham (all *P* > 0.05).

### 3.5 ES upregulates neuroprotective gene and protein expression in the degenerating retina

Based on the functional and structural outcomes described in Sections 3.2–3.4, TpES at 800 µA and TbES at 50 Hz showed the most pronounced effects among the tested parameters within their respective modalities—yielding the highest ERG amplitudes, greatest retinal thickness preservation, and lowest apoptotic indices across the two stimulation paradigms. TpES at 600 µA and TbES at 100 Hz were additionally selected as adjacent parameters to enable dose–response characterization and to determine whether the molecular effects were specific to the optimal parameters or were shared more broadly within each parameter.

**FIGURE 5:**
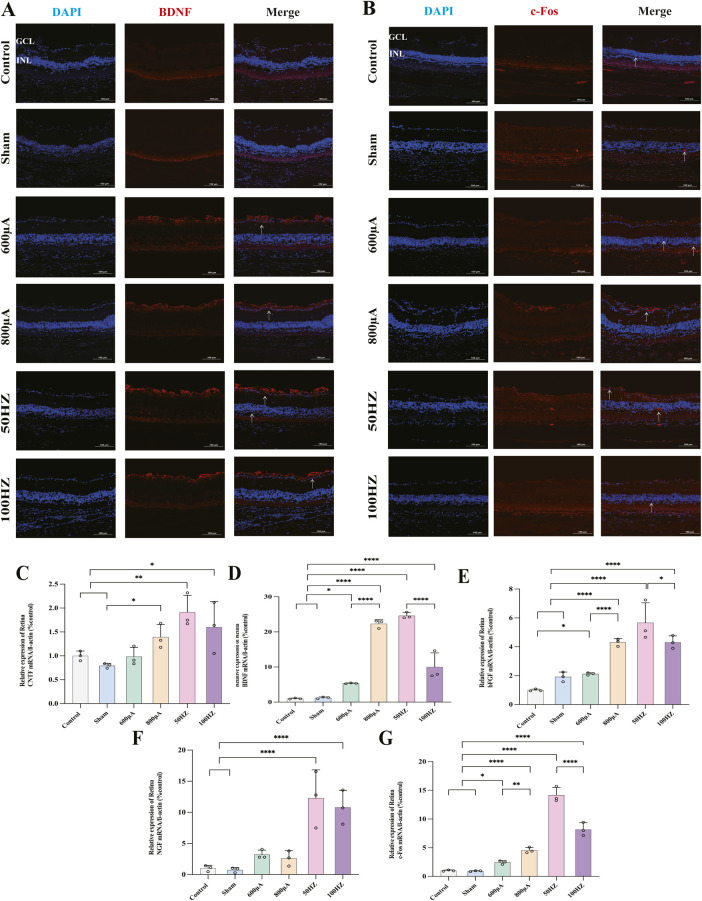
Gene and protein expression analysis of neuroprotective factors in RCS rat retinas at 14 days post-stimulation. **(A)** Representative IF images of BDNF (red) and DAPI (blue) in retinal cross-sections from all experimental groups (Control, Sham, TpES: 600 µA, 800 µA; TbES: 50 Hz, 100 Hz). BDNF immunoreactivity is predominantly localized to the INL and GCL. **(B)** Representative IF images of c-Fos (red) and DAPI (blue) in retinal sections from the same groups. c-Fos expression is concentrated within the INL and GCL, consistent with its role as a marker of inner retinal neuronal activation. The white arrow indicates a positive expression signal. Scale bar = 100 µm. Relative mRNA expression of **(C)** CNTF, **(D)** BDNF, **(E)** b-FGF, **(F)** NGF, and **(G)** c-Fos in retinal tissue, normalized to β-actin and expressed as fold-change relative to the untreated Control group. The horizontal brackets indicate that the grouped items within are statistically different from the other group. Data are presented as mean ± SD (n = 3). GCL: ganglion cell layer; INL: inner nuclear layer; IF: immunofluorescence. *P < 0.05, **P < 0.01, ***P < 0.001, ****P < 0.0001.

To validate these mRNA findings at the protein level, IF staining was performed to assess the spatial distribution and relative expression of BDNF and c-Fos in retinal sections at 14 days post-stimulation (Figures 5A,B). BDNF immunoreactivity (red fluorescence) was predominantly localized to the inner nuclear layer (INL) and ganglion cell layer (GCL), displaying a continuous band-like distribution consistent with the retinal laminar architecture as delineated by DAPI counterstaining. BDNF fluorescence signal was minimal in the Control group, and remained comparably low in the Sham group, indicating that handling alone does not produce a detectable protein-level increase. Stimulated groups showed progressive increases in BDNF signal intensity, accompanied by a broadening of the immunoreactive band. The 800 µA TpES and 50 Hz TbES groups exhibited the most prominent fluorescence, and the 100 Hz TbES group maintained comparably elevated immunoreactivity. c-Fos immunoreactivity was similarly concentrated within the INL and GCL, consistent with its role as an immediate early gene marker of neuronal activity. c-Fos fluorescence was virtually absent in both Control and Sham groups, confirming that neither disease progression nor the handling procedure alone was sufficient to activate inner retinal neurons. c-Fos signal increased progressively with stimulation, with the 50 Hz TbES and 800 µA TpES groups displaying the strongest and most densely distributed expression.

As shown in Figures 5C–G, most relative expressions were significantly upregulated in CNTF, BDNF, FGF, NGF, and c-Fos in the 800 μA and 50 Hz group. No significant difference was observed between the Sham and Control groups in all targeted genes, indicating that Isoflurane and non-current stimulation did not alter basal expression. The 50 Hz (1.91 ± 0.35) and 100HZ (1.60 ± 0.53) groups demonstrated a statistically significant increase in retinal CNTF mRNA relative to both Control (1.00 ± 0.10, *P* < 0.05) and Sham (0.79 ± 0.05, *P* < 0.01; Figure 5C); The 800 µA TpES group (1.39 ± 0.26) showed a trend toward elevation but reached significance only against the Sham group (*P* < 0.05). BDNF mRNA expression was markedly upregulated in all ES groups relative to both reference groups (Figure 5D). The most pronounced induction was observed in the 50 Hz group (24.63 ± 0.88, *P* < 0.0001), followed closely by the 800 µA group (22.32 ± 1.17, *P* < 0.0001), representing approximately 22–25-fold increases over Sham (1.23 ± 0.25) and Control (1.00 ± 0.16) groups. A consistent upregulation of b-FGF mRNA was detected across all stimulated groups compared with Sham (1.92 ± 0.34) and Control (1.00 ± 0.07; Figure 5E). The 50 Hz group (5.67 ± 1.38) exhibited the highest b-FGF expression (*P* < 0.0001), followed by the 800 µA group (4.31 ± 0.25, *P* < 0.0001) and the 100 Hz TbES group (4.32 ± 0.45, *P* < 0.0001). NGF mRNA levels were significantly elevated in the 50 Hz TbES (12.28 ± 4.55, *P* < 0.0001) and 100 Hz TbES groups (10.80 ± 2.73, *P* < 0.0001) relative to Sham (0.73 ± 0.40) and Control (1.01 ± 0.49; Figure 5F). Neither TpES group replicated this pattern, confirming that robust NGF induction is preferentially associated with TbES delivery. C-Fos mRNA expression showed a pronounced, highly significant upregulation in both TbES groups relative to Sham (0.93 ± 0.85) and Control (1.00 ± 0.12; Figure 5G). The 50 Hz group (14.17 ± 1.34, *P* < 0.0001) showed the greatest induction, followed by the 100 Hz group (8.19 ± 1.16, *P* < 0.0001). Among TpES groups, the 800 µA condition significantly elevated c-Fos expression (4.55 ± 0.50; *P* < 0.0001), while the 600 µA group (2.51 ± 0.32) also reached significance against both reference groups (*P* < 0.05).

## 4 Discussion

This study presents the neuroprotective effects of two non-invasive ES methods, TpES and TbES, when applied to acupoints along the Gallbladder Meridian in the RCS rat model of hereditary retinal degeneration. Our results demonstrate that both modalities confer significant neuroprotection in a parameter-dependent manner: TpES at 800 µA and TbES at 50 Hz consistently yield the greatest preservation of retinal function and structural integrity, reduction in photoreceptor apoptosis, and upregulation of neuroprotective gene and protein expression. These results defined unique, optimized ES parameters for rescuing photoreceptors and visual function in RP. It should be emphasized that 800 μA and 50 Hz were each identified as the optimal parameter within their respective modality (TpES and TbES), rather than as statistically equivalent conditions.

OFT assesses animals’ activity, exploration, and anxiety; vision is essential for spatial navigation (DeFries et al., 1966; Berry et al., 2019). Mice avoid open spaces and stay near walls. In this study, Untreated control rats exhibited a progressive increase in total distance moved (+38.9%) and cumulative central-zone duration (+80%) between 7 and 14 days. While changes of this character have been reported in rodent models of retinal degeneration and attributed to anxiety-like behavior secondary to progressive vision loss (Hann Yih et al., 2022), the open-field paradigm cannot unambiguously exclude alternative contributors—including pain, cumulative handling stress, or residual anaesthetic effects—as underlying causes. Conversely, the TpES (400–800 µA) and TbES (15–100 Hz) groups maintain periphery-dominant exploration patterns comparable to the sham group, with no differences in velocity, distance, or center duration (*P* > 0.05), suggesting that the stimulation protocols did not adversely affect general locomotor behavior. The 200 µA TpES group exhibited fewer zone transitions, yet speed and distance remained unchanged; this low-intensity condition conferred the weakest retinal protection. This finding likely reflects reduced exploratory drive from lower anxiety rather than motor impairment—supported by Berry et al., who reported that animals with restored vision perform more efficient, goal-directed exploration in the open field (Berry et al., 2019). Additionally, a recent study showed that effective neuroprotective interventions do not impair general locomotor activity (Ma et al., 2025). Overall, these results indicate that optimized electrical stimulation was not associated with overt behavioral abnormalities, but the OFT findings should be regarded as supportive and indirect rather than as direct evidence of visual recovery.

There is increasing evidence that non-invasive ES of the eye holds therapeutic potential across various retinal diseases, including RP (Morimoto et al., 2007; Morimoto et al., 2012; Hanif et al., 2016; Tao et al., 2016; Yu et al., 2020; Liu et al., 2022), retinal degeneration (Ni et al., 2009; Schatz et al., 2012; Agadagba et al., 2022; Lennikov et al., 2025), high intraocular pressure injury (Wang et al., 2011; Fu et al., 2018; Jassim et al., 2021), traumatic optic neuropathy (Morimoto et al., 2005; Morimoto et al., 2010; Miyake et al., 2007; Tagami et al., 2009; Henrich-Noack et al., 2013a; Henrich-Noack et al., 2013b; Henrich-Noack et al., 2017; Yin et al., 2016), and non-arteritic ischemic optic neuropathy (Osako et al., 2013). In the present study, ERG analysis revealed that TpES at 800 µA and TbES at 50 Hz provided maximal protection for scotopic and photopic responses at both 7 and 14 days, underscoring the critical role of parameter selection. SD-OCT and HE staining confirmed that the optimal-parameter groups maintained significantly greater total retinal and ONL thickness at 14 days, with 3–4 photoreceptor cell layers preserved compared with 1–2 in sham and control retinas. These findings are consistent with previous research showing that ES can lessen photoreceptor loss in models of retinal degeneration, including transcorneal ES in RCS rats (Morimoto et al., 2007) and transscleral ES in rd10 mice (Liu et al., 2022). These findings contribute to a growing body of literature aimed at defining optimal stimulation windows. In the context of RGC protection following optic nerve transection, Morimoto et al. established a distinct set of optimal parameters (100–200 mA, 1–3 ms/phase, 1–20 Hz), suggesting that requirements for RGC survival may differ from those for photoreceptor preservation (Morimoto et al., 2010). Comparatively, addressing outer retinal degeneration, Enayati et al. found that in Rho−/− mice, a more complex, low-current waveform (100 µA with combined rectangular and ramp sequences) was particularly effective in enhancing photoreceptor survival and bipolar cell connectivity (Enayati et al., 2024). Taken together, these studies-including our own-highlight that the therapeutic efficacy of noninvasive ES is not only parameter-specific but also likely contingent upon the targeted retinal cell type and the underlying disease model.

ES confers neuroprotection through dual mechanisms: inhibiting photoreceptor apoptosis and upregulating neurotrophic factor cascades. TUNEL staining further quantifies the anti-apoptotic effect: TpES at 800 µA yields the greatest reduction in apoptotic index (∼0.35 vs. controls), while all TbES frequencies lower the apoptotic index to a similar range (0.39–0.40), indicating a frequency-independent anti-apoptotic threshold for TbES delivery. At the molecular level, both TpES and TbES upregulated BDNF expression in the INL and GCL, consistent with its established role in ES-induced retinal neuroprotection (Sato et al., 2008). The Müller cells, which maintain structural and functional stabilities of retinal neurons, are the primary cellular source of ES-induced BDNF, CNTF, bFGF, and IGF-1 (Chang et al., 2021). Notably, TbES—particularly at 50 Hz—elicited a broader neurotrophic response, with significant upregulation of CNTF (50 Hz only) and NGF (50 and 100 Hz), a pattern not observed in any TpES group. Previous studies have reported that transcorneal ES protects photoreceptors by upregulating CNTF and BDNF in an N-methyl-N-nitrosourea-induced degeneration model (Tao et al., 2016). However, in our RCS model, this synergistic effect was observed only with TbES, not with TpES. We speculate that this discrepancy may reflect differences in the cellular microenvironment, the underlying disease etiology (inherited MERTK mutation vs. chemical insult), and the distinct neural pathways engaged by transcranial versus periocular stimulation. Elevated c-Fos expression in the INL and GCL—confirmed by both IF and dPCR—further indicates activation of inner retinal neurons. Given that TbES targets the visual cortex via Gallbladder meridian acupoints, this upregulation may reflect broader neuronal activation along the retinocortical axis, consistent with reports that transcorneal ES activates c-Fos–labeled neurons in brain sections (Gomez-Maldonado et al., 2025).

Vision-perception is formed and maintained through the structural integrity and functional integration of the retina-visual pathway (Banna et al., 2024). While isolated interventions on the peripheral retina may alleviate local lesions, the coordinated regulation of vision-related brain regions is particularly important for achieving systemic improvements in visual function and central plasticity (London et al., 2013). Interestingly, TCM has long recognised the close connection between the head meridians and the functions of the eyes; in particular, the Foot Shaoyang Gallbladder Meridian, which originates at the inner canthus of the eye, passes through several key acupoints for treating eye conditions. The selection of acupoints (GB14, GB16, and GB20) in this study is grounded in both TCM theory and our prior clinical experience. Our group has demonstrated the safety and feasibility of TpES in patients with RP, providing the empirical foundation for the integrated eye–brain approach developed in the future study (Zhou et al., 2024). TpES delivers direct, well-tolerated noninvasive ES, while TbES at GB14, GB16, and GB20 engages broader cortical networks along the full visual axis, potentially extending neuroprotection upstream to the lateral geniculate nucleus and primary visual cortex. Integrating Chinese and Western medicine in this way helps to regulate the excitability and connectivity of vision-related brain networks, thereby supporting the overall rehabilitation of visual function.

Several limitations should be acknowledged. First of all, the 14-day observation period, while sufficient to capture protective effects during the peak degeneration phase in RCS rats, does not address the durability of these benefits; extended longitudinal studies are needed. Then, the RCS rat model, driven by a single MERTK mutation, may not fully represent the genetic and phenotypic heterogeneity of human RP. Future validation in additional models (e.g., P23H rats, rd10 mice) would strengthen translatability. Third, the OFT test is not a vision-specific assay. Future studies should incorporate validated vision-specific behavioral assays such as the optomotor response test or Morris water maze. Fourth, the relatively small sample size limits the detection of smaller or moderate effects, and non-significant comparisons should be interpreted cautiously as they may reflect low statistical power rather than absence of biological effect. Moreover, although both male and female rats were included, sex-stratified analysis was not feasible due to insufficient subgroup sample sizes, leaving the potential influence of sex undetermined. Given the exploratory, multi-parameter nature of this study, increasing animal numbers would have conflicted with the 3R principle; thus, our findings should be considered preliminary and hypothesis-generating, and confirmatory studies with larger, adequately powered and sex-balanced cohorts are needed. Furthermore, this study focused on a limited set of neurotrophic factors, a more comprehensive mechanistic analysis, including anti-inflammatory cytokines, apoptosis regulators, and energy metabolism markers—was not performed and would provide deeper insight into the pathways engaged by TpES and TbES. Last but not least, this study did not evaluate concurrent TpES and TbES intervention, a logical next step given the complementary retinal and cortical targets of the two modalities.

In summary, this study shows that optimized TpES and TbES have significant neuroprotective effects in the RCS rat model of retinal degeneration, preserving function, structure, reducing apoptosis, and upregulating neurotrophic factors. TpES at 800 µA and TbES at 50 Hz were identified as optimal parameters within their respective modalities. These findings provide evidence-based parameters for clinical use and lay the groundwork for future trials of multi-modal ES as a safe, accessible, and effective treatment for RP and retinal dystrophies.

## Data Availability

The raw data supporting the conclusions of this article will be made available by the authors, without undue reservation.
