# Supplementary material for: Optimizing stimulation parameters: transpalpebral and transbrain electrical stimulation for retinal protection in RCS rats
Source: Front Cell Dev Biol. 2026 Jul 17;14:1853872. doi: 10.3389/fcell.2026.1853872 (PMC13423766; doi:10.3389/fcell.2026.1853872)
Supplement: Supplementary file 1 [file Table1.docx]

**Supplementary Table 1. Charge per phase and charge density per phase for TpES and TbES**

| **Group** | **Frequency** | **Current** | **Phase width (ms)** | **Charge per phase (µC/phase)** | **Charge density per phase (µC/cm²/phase)** |
| --- | --- | --- | --- | --- | --- |
| TpES 200 µA | 292 Hz | 200 µA | 0.21 | 0.042 | 0.116 |
| TpES 200 µA | 30 Hz | 200 µA | 0.56 | 0.112 | 0.308 |
| TpES 200 µA | 9.1 Hz | 200 µA | 0.59 | 0.118 | 0.325 |
| TpES 200 µA | 0.3 Hz | 200 µA | 0.60 | 0.12 | 0.33 |
| TpES 400 µA | 292 Hz | 400 µA | 0.21 | 0.084 | 0.231 |
| TpES 400 µA | 30 Hz | 400 µA | 0.56 | 0.224 | 0.617 |
| TpES 400 µA | 9.1 Hz | 400 µA | 0.59 | 0.236 | 0.65 |
| TpES 400 µA | 0.3 Hz | 400 µA | 0.60 | 0.24 | 0.66 |
| TpES 600 µA | 292 Hz | 600 µA | 0.21 | 0.126 | 0.347 |
| TpES 600 µA | 30 Hz | 600 µA | 0.56 | 0.336 | 0.825 |
| TpES 600 µA | 9.1 Hz | 600 µA | 0.59 | 0.354 | 0.975 |
| TpES 600 µA | 0.3 Hz | 600 µA | 0.60 | 0.36 | 0.991 |
| TpES 800 µA | 292 Hz | 800 µA | 0.21 | 0.168 | 0.463 |
| TpES 800 µA | 30 Hz | 800 µA | 0.56 | 0.448 | 1.233 |
| TpES 800 µA | 9.1 Hz | 800 µA | 0.59 | 0.472 | 1.23 |
| TpES 800 µA | 0.3 Hz | 800 µA | 0.60 | 0.480 | 1.322 |
| TbES 15 Hz | 15 Hz | 1 mA | 0.58 | 0.580 | 73.848 |
| TbES 30 Hz | 30 Hz | 1 mA | 0.56 | 0.560 | 71.301 |
| TbES 50 Hz | 50 Hz | 1 mA | 0.53 | 0.530 | 67.482 |
| TbES 100 Hz | 100 Hz | 1 mA | 0.47 | 0.470 | 59.842 |

**Supplementary note :**Charge per phase was calculated as *Q=I×PW*, where I is the stimulation current and PW is the phase width. To express charge in µC, the product of current (µA) and phase width (ms) was divided by 1000. Charge density per phase was calculated as *Q/A*, where A is the geometric electrode area. The TpES surface electrode had a radius of 3.4 mm (area = 0.3632 cm²), and the TbES needle electrode had a radius of 0.5 mm (area = 0.00785 cm²).
