# Supplementary material for: Optimizing stimulation parameters: transpalpebral and transbrain electrical stimulation for retinal protection in RCS rats
Source: Front Cell Dev Biol. 2026 Jul 17;14:1853872. doi: 10.3389/fcell.2026.1853872 (PMC13423766; doi:10.3389/fcell.2026.1853872)
Supplement: Supplementary file 2 [file DataSheet1.docx]

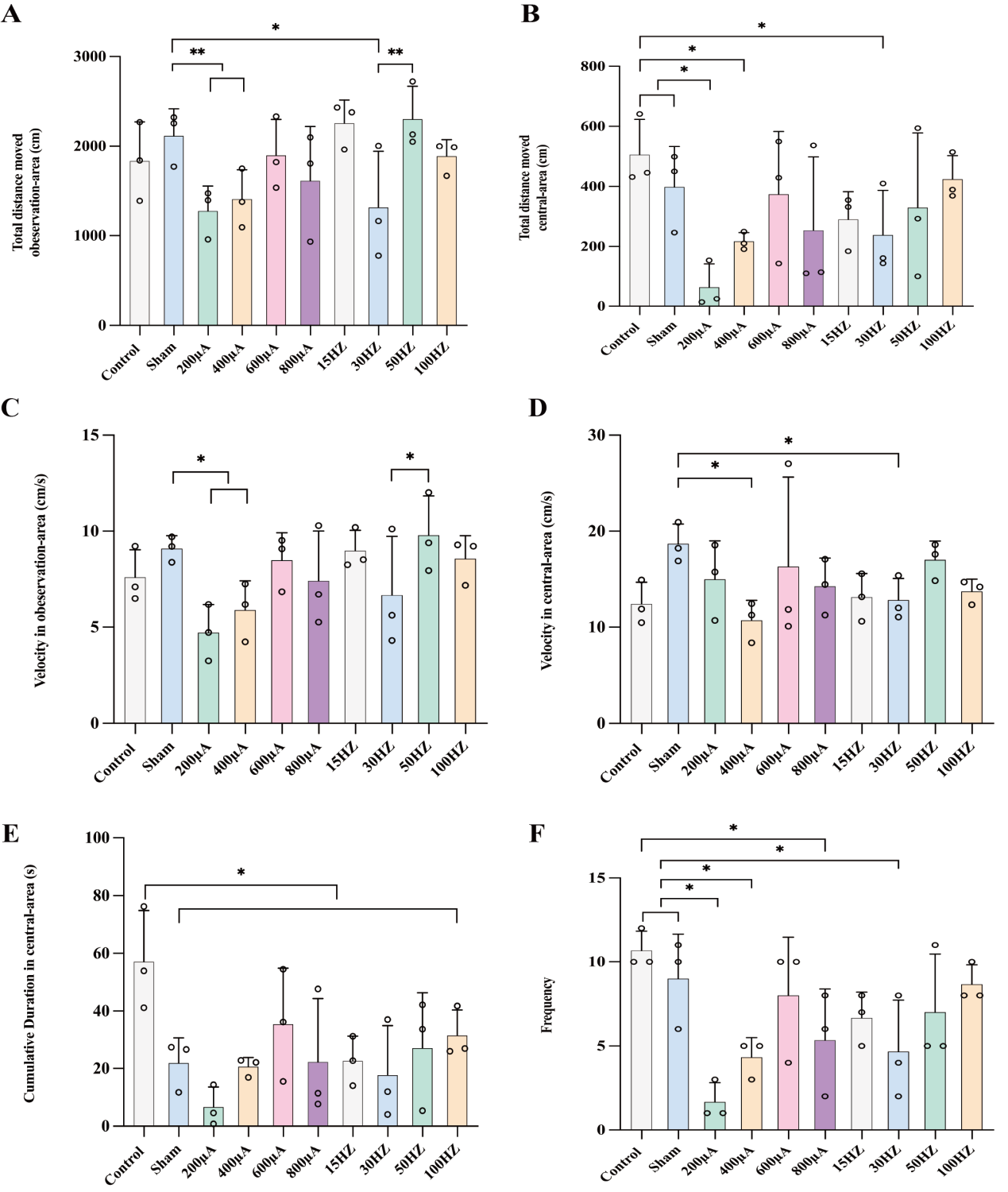


**Supplementary Figure 1 Effects of TpES and TbES on open field test behavior in RCS rats at 14 days post-stimulation. (A) Total distance moved in the observation area at 14d. (B) Total distance moved in the central area at 14d. (C) Mean velocity in the observation area at 14d. (D)** **Mean velocity in the central area at 14d. (E) Center-point cumulative duration at 14d. (F) Zone transition frequency to the central area at 14d. The horizontal brackets indicate that the grouped items within are statistically different from the other group. Data are presented as mean ± SD (n = 3). **P* < 0.05, ***P* < 0.01.**


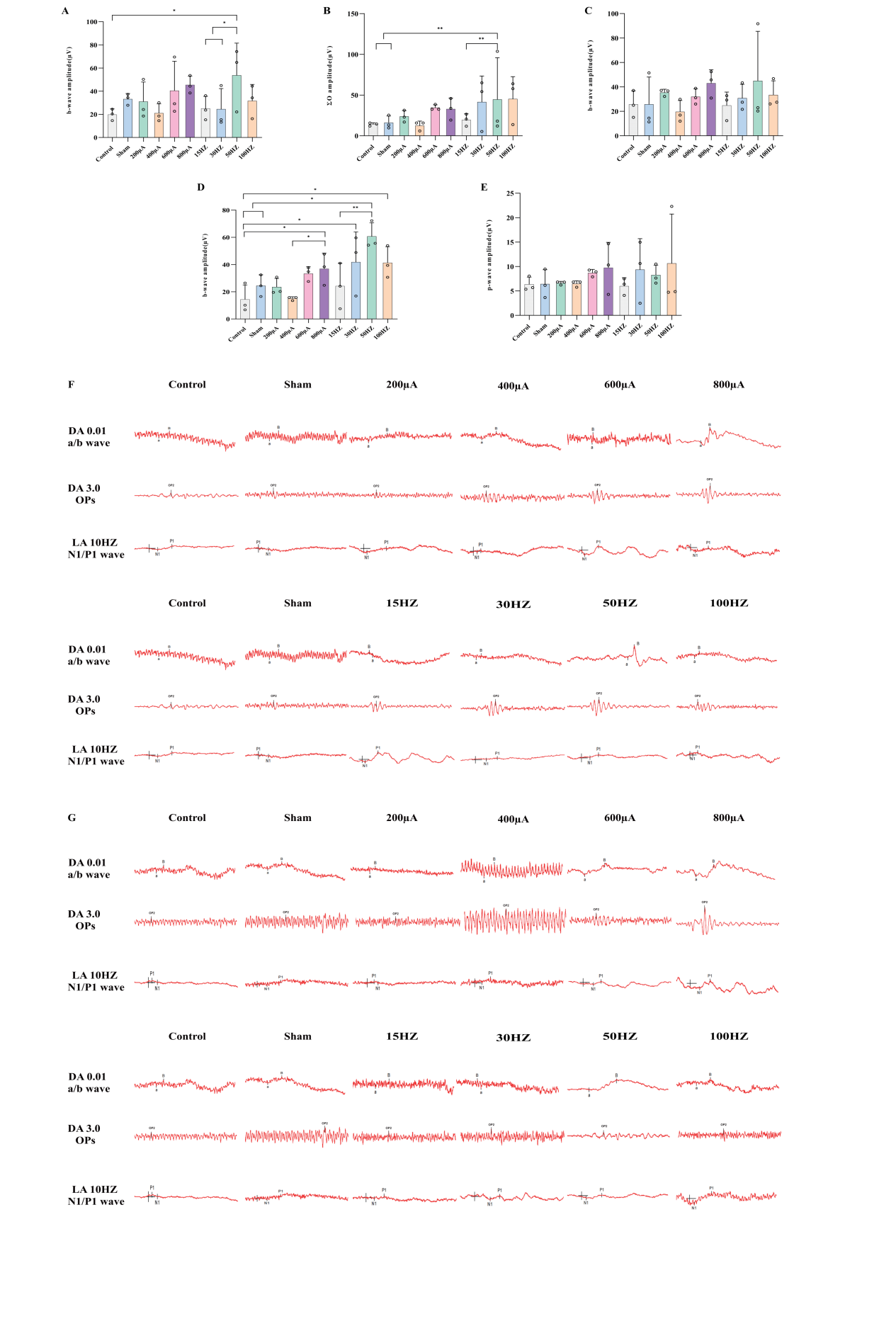


**Supplementary Figure 2 ERG response amplitudes at 14 days post-treatment across all experimental groups.**

(A) Scotopic rod b-wave amplitude. (B) Oscillatory potentials (OPs, expressed as ΣO amplitude). (C) Maximal mixed response b-wave amplitude. (D) Photopic cone b-wave amplitude. (E) 10 Hz flicker p-wave amplitude. (F) Representative scotopic ERGs (0.01 cd·s/m2), oscillatory potentials (Ops) (3.0 cd·s/m2), and photopic ERGs (10.0 cd·s/m2) are shown for each experimental group at 7 days. Groups: Control, Sham, TpES (upper), TbES (down). (G) Representative scotopic ERGs, oscillatory potentials (Ops), and photopic ERGs are shown for each experimental group at 14 days. Each trace is a single recording from one eye, selected as typical for that group. The horizontal brackets indicate that the grouped items within are statistically different from the other group. Data are presented as mean ± SD (n = 3). **P* < 0.05, ***P* < 0.01, ****P* < 0.001, *****P* < 0.0001.


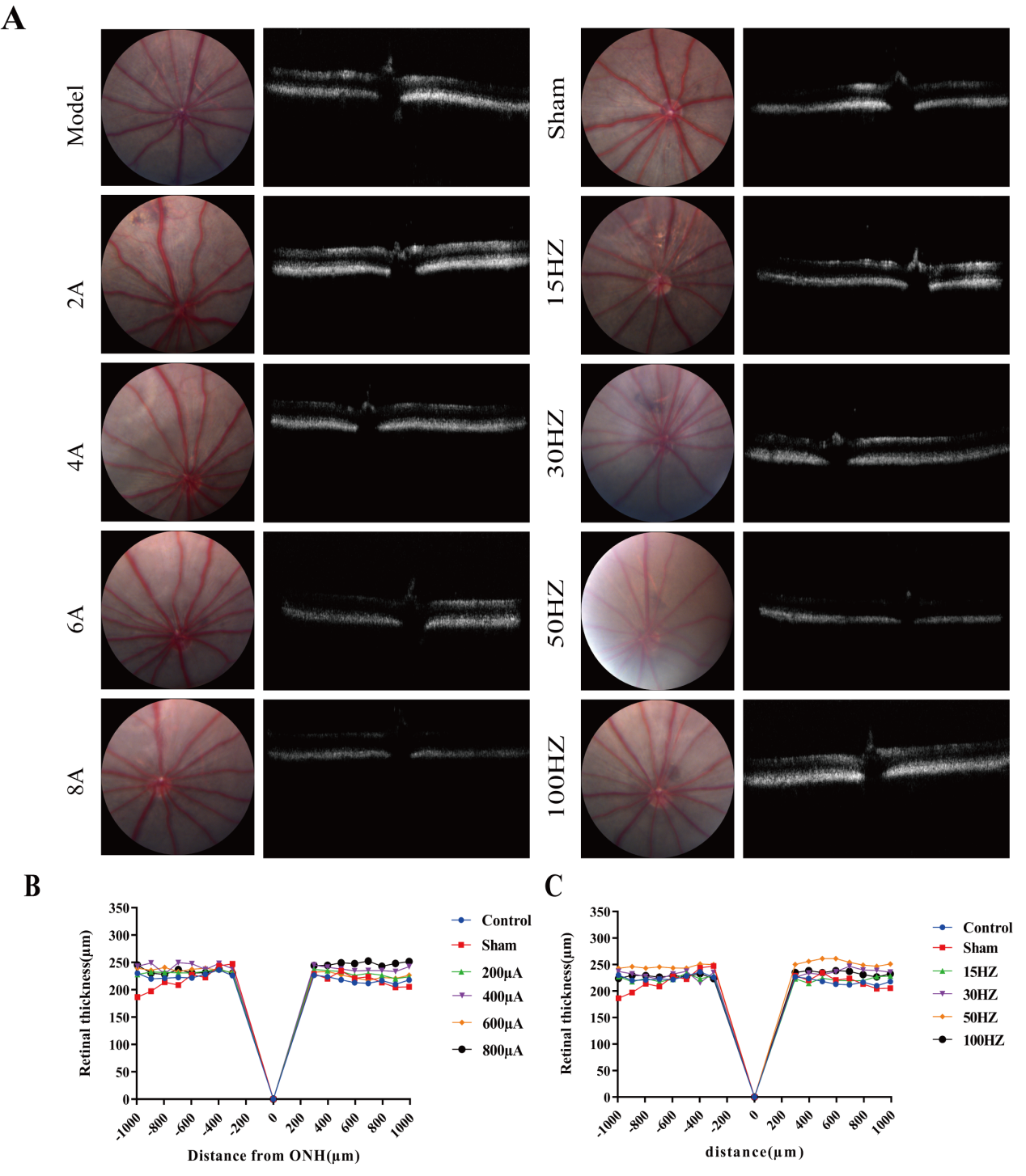


**Supplementary Figure 3 Retinal morphology in RCS rats following ES at varying parameters.**

(A) Representative CFP (left) and SD-OCT cross-sectional images (right) of TpES groups (Control, 200, 400, 600, 800 µA) and TbES groups (Sham, 15, 30, 50, 100 Hz) at 14 days post-stimulation. (B) Quantitative retinal thickness along the ventral–dorsal meridian for TpES groups at 14 days post-stimulation. (C) Quantitative retinal thickness along the ventral–dorsal meridian for TbES groups at 14 days post-stimulation. Data are presented as mean ± SD (n = 3).
